# Interspecific variation in the diet of *Symphalangussyndactylus* and *Macacanemestrina* at Genting Highlands, Pahang, Peninsular Malaysia

**DOI:** 10.3897/BDJ.12.e122453

**Published:** 2024-05-22

**Authors:** Roberta Chaya Tawie Tingga, Millawati Gani, Nur Azimah Osman, Nor Rahman Aifat, Eddie Chan, Shamsul Khamis, Emelda Rosseleena Rohani, Norlinda Mohd-Daut, Abd Rahman Mohd-Ridwan, Badrul Munir Md-Zain

**Affiliations:** 1 Department of Biological Sciences and Biotechnology, Faculty of Science and Technology, Universiti Kebangsaan Malaysia, Bangi, 43600, Selangor, Malaysia Department of Biological Sciences and Biotechnology, Faculty of Science and Technology, Universiti Kebangsaan Malaysia Bangi, 43600, Selangor Malaysia; 2 Centre for Pre-University Studies, Universiti Malaysia Sarawak, 94300, Kota Samarahan, Sarawak, Malaysia Centre for Pre-University Studies, Universiti Malaysia Sarawak, 94300 Kota Samarahan, Sarawak Malaysia; 3 National Wildlife Forensic Laboratory (NWFL), Department of Wildlife and National Parks (PERHILITAN), KM 10 Jalan Cheras, 56100 Kuala Lumpur, Malaysia National Wildlife Forensic Laboratory (NWFL), Department of Wildlife and National Parks (PERHILITAN), KM 10 Jalan Cheras 56100 Kuala Lumpur Malaysia; 4 School of Biology, Faculty of Applied Sciences, Universiti Teknologi Mara Negeri Sembilan, 72000, Kuala Pilah, Negeri Sembilan, Malaysia School of Biology, Faculty of Applied Sciences, Universiti Teknologi Mara Negeri Sembilan, 72000 Kuala Pilah, Negeri Sembilan Malaysia; 5 Faculty of Tropical Forestry, Universiti Malaysia Sabah, 88400, Kota Kinabalu, Sabah, Malaysia Faculty of Tropical Forestry, Universiti Malaysia Sabah, 88400 Kota Kinabalu, Sabah Malaysia; 6 Genting Nature Adventure, Resorts World Awana Hotel, 69000, Genting Highlands, Pahang, Malaysia Genting Nature Adventure, Resorts World Awana Hotel, 69000, Genting Highlands Pahang Malaysia; 7 Institute of Systems Biology, Universiti Kebangsaan Malaysia, Bangi, 43600, Selangor, Malaysia Institute of Systems Biology, Universiti Kebangsaan Malaysia Bangi, 43600, Selangor Malaysia

**Keywords:** Cercopithecidae, diet, DNA metabarcoding, Hylobatidae, interspecific competition

## Abstract

Primate communities in the Genting Highlands consist of a single species of Hylobatidae and four species of Cercopithecidae, which are known to exhibit social interaction behaviour. Thus, a study on the diets of *Symphalangussyndactylus* (siamang; family Hylobatidae) and *Macacanemestrina* (pig-tailed macaque; family Cercopithecidae) was carried out at Genting Highlands, in order to compare the dietary preferences and interspecific competition between the two primate families. A DNA metabarcoding approach was used to analyse diet intake using non-invasive samples based on the trnL region. Based on the 140 amplicon sequence variants (ASVs) generated, 26 plant orders, 46 different families, 60 genera and 49 species were identified from 23 different plant classes. Fabaceae and Moraceae were classified as the most preferred plants at the family level for *S.syndactylus*; meanwhile, Piperaceae and Arecaceae were classified as the most preferred for *M.nemestrina*. Only six out of the 60 different plant genera classified in this study, were found to be consumed by both species. Therefore, the low similarity of preferred plants in the diets between the two families suggests that there is little interspecific competition. These findings are important for future conservation management of highland primates, especially in the Genting Highlands.

## Introduction

Genting Highlands is a potential highland area for ecotourism, with a height of 1770 m a.s.l. and has several residential neighbourhood hotels, amusement parks and casinos at the top (Peh et al. 2011). It is located in Bentong, Pahang. Despite the hilltop of the Genting Highlands being opened for amusement and tourism activities, approximately 4297 ha of the surrounding logged forest is being protected for sustainability purposes (Peh et al. 2011). The foothills and peaks of Gunung Bunga Buah and Gunung Ulu Kali have been identified as suitable flora conservation sites (Ariffin and Kumari 1989, Kumari 1989). The area is predicted to have over 460 types of flowering and non-flowering plants, divided into 90 families. In terms of fauna diversity, the Genting Highlands has diverse wildlife, including 18 amphibian, 134 bird, 42 mammal and 18 reptile species (Musthafa and Abdullah 2019). Nevertheless, the continuous rapid development of the surrounding area in the Genting Highlands has impacted the forest area, thereby altering the composition of flora and fauna species (Chua and Saw 2001). Chua and Saw (2001) also noted a dramatic decrease in floral variations in the Genting Highlands because of significant environmental changes.

**Figure 1.**
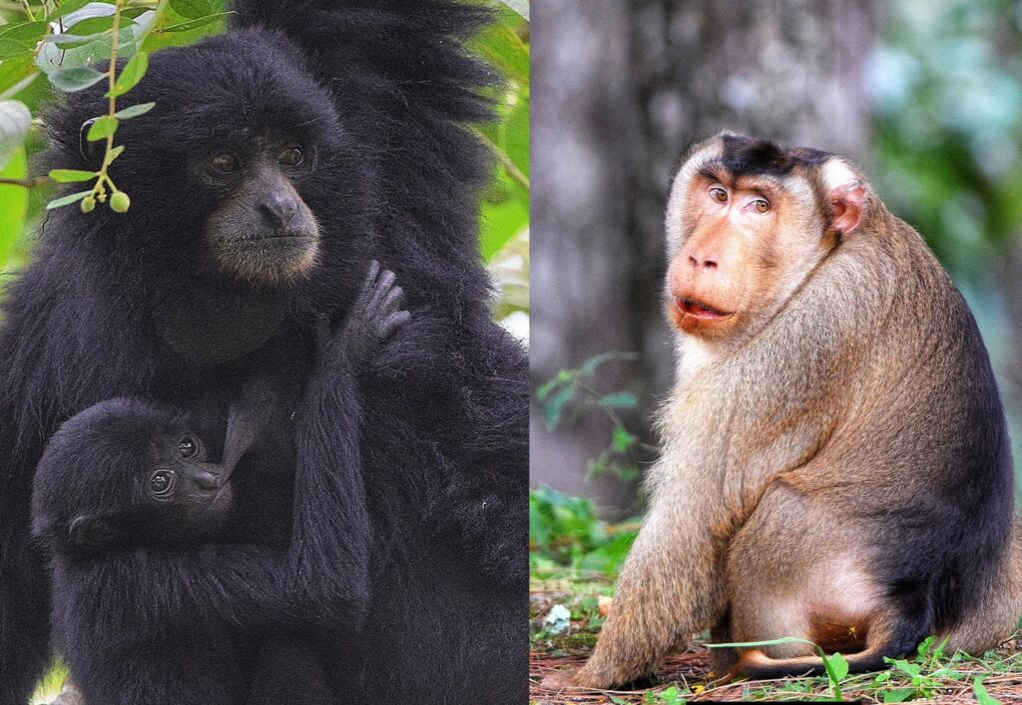
*Symphalangussyndactylus* (siamang) (left) and *Macacanemestrina* (pig-tailed macaque) (right) of Genting Highlands. Photo by Eddie Chan.

Early reports have identified a few species of primates in the Genting Highlands, including *Symphalangussyndactylus*, *Trachypithecusobscurus*, *Presbytissiamensis*, *Macacanemestrina* and *Macacafascicularis* (Md-Zain et al. 2021). Conducting studies on primate species within the Genting Highlands is suitable because it provides an excellent habitat for primates with a relatively preserved area, free from logging activities that could obstruct the primate’s access to food or shelter (Azmi 2021). Furthermore, the occurrence of mixed-species associations within the primate communities in the Genting Highlands was previously observed to have social interaction with each other in close proximity (Md-Zain et al. 2021). Based on these results, it was also stated that *S.syndactylus* exhibited non-hostile interactions since it made an effort to get close to other species, including *M.fascicularis* and *T.obscurus*. An interaction that involves any two or more species that live in close proximity is referred to as an interspecific association. These interactions can be either direct or indirect and are characterised by aggressive behaviour, competition or territoriality (Levins 1979, Armstrong and McGehee 1980). Thus, it is interesting to observe the possibility of interspecific competition on diet between different families of primates in the Genting Highlands. This study focused only on two selected species, *S.syndactylus* (siamang) and *M.nemestrina* (pig-tailed macaque), which represent the families Hylobatidae and Cercopithecidae, respectively (Fig. 1).

*Symphalangussyndactylus* (siamang) is the largest member of the family Hylobatidae and can be found in the Malay Peninsula and Sumatra. There are two recognised subspecies: *S.syndactylus* (Sumatra) and *S.syndactyluscontinents* (Malay Peninsular) (Gron 2008). A recent study by Sariyati et al. (2024) also reported that, based on the hypervariable region of mitochondrial DNA, molecular phylogeny validates the subspecies delineation of the Malayan Siamang (*Symphalangussyndactyluscontinentis*) and the Sumatran Siamang (*Symphalangussyndactylussyndactylus*). *Symphalangussyndactylus* can easily manoeuvre through trees by performing hand-over-hand movements with a swinging posture because they have long arms and well-adapted hands. One of the distinguishing features of *S.syndactylus* that differentiates them from other gibbons is their distinctively loud call (Marshall and Sugardjito 1986). Both males and females will assist in synchronising their calls to mark their territory. They can be found in lowland areas up to 1,500 m a.s.l. in primary and secondary forests (Shepherd and Shepherd 2017). In terms of their diet, *S.syndactylus* generally consumes most parts of plants, including young leaves (Nurcahyo 2001), fruits, leaves (Macdonald 2004) and bark (Riley 2008). Currently, *S.syndactylus* is classified as an endangered species according to the International Union for Conservation of Nature (IUCN 2022). Habitat loss and habitat degradation are the main threats to the population of *S.syndactylus* (Nijman et al. 2020).

*Macacanemestrina*, known as the Southern pig-tailed macaque, is classified under the family Cercopithecidae. The distribution of *M.nemestrina* includes Peninsular Malaysia, Borneo, Sumatra, Bangka and Thailand (Roos et al. 2014). Locals from Penang Island on the west coast and Tioman Island on the east coast of Peninsular Malaysia refer to them as "beruk" (Abdul-Latiff et al. 2014, Roos et al. 2014, Abdul-Latiff and Md-Zain 2021). *Macacanemestrina* is closely associated with rainforest habitats. They are also commonly found in hilly areas (Shepherd and Shepherd 2017) and primarily forage on the ground and their diet includes fruits and small insects (Shepherd and Shepherd 2017). Previously, there were three subspecies of *M.nemestrina*, namely *M.nemestrinaleonina*, *M.nemestrinapagensis* and *M.nemestrinanemestrina* (Fooden 1975). These three subspecies were previously recognised, based on morphological characteristics, which are pelage colour, pattern and tail morphology. Roos et al. (2014) later amended the subspecies classification and assigned them according to the species level. In a recent study by Abdul-Latiff and Md-Zain (2021), a new subspecies of *M.nemestrina*, namely *M.nemestrinaperakensis*, was proposed and named, based on their location in Selama, Perak. The new subspecies was described, based on its morphology and genetics, which differ from *M.nemestrinanemestrina*. At present, *M.nemestrina* is listed as endangered by the IUCN and its population is primarily decreasing because of habitat loss, particularly widespread deforestation and the degradation of lowland forests in Malaysia. Other threats to *M.nemestrina* include hunting activities and the illegal pet trade (IUCN 2022).

One of the novel molecular methods to compare and assess the interspecific variation in diet between *S.syndactylus* and *M.nemestrina* efficiently is DNA metabarcoding. DNA metabarcoding is a recent technological advancement that allows diet assessment in primates to be performed using non-invasive techniques via faecal collection. This technique is particularly beneficial when direct observation of feeding is not possible, especially for species that are free and wild and where plants are required to be visually identified. One of the advantages is that it provides better coverage when identifying rare taxa within an ecosystem (Pavan-Kumar et al. 2015) by generating hundreds of thousands or millions of sequencing reads to identify species (Othman et al. 2021). DNA metabarcoding is able to perform plant dietary analyses (Mallott et al. 2018) and the use of the trnL gene is a more robust marker than other markers such as rbcL. Its P6 loop region is sufficient to perform DNA metabarcoding on a degraded sample, such as a faecal sample (Taberlet et al. 2007). In recent years, there have been a few mammal diet studies that have successfully used the DNA metabarcoding approach in Malaysia, such as Osman et al. (2020), Osman et al. (2022) [primate], Abdullah-Fauzi et al. (2022) and Mohd-Radzi et al. 2022 [elephant]. This implies that DNA metabarcoding offers a wider and more powerful diet assessment compared to direct observation. This technique has also proven successful in being widely used in other aspects, such as microbiome studies (Mohd-Yusof et al. 2022, Gani et al. 2024).

At present, there is a lack of studies considering the diet of wild *S.syndactylus* and *M.nemestrina*, especially in highland areas, such as the Genting Highlands. Genting Highlands is a suitable site to conduct research on primate diets because it is surrounded by both developed regions, including hotels and housing estates and agricultural areas, as well as the remaining forest (Ng et al. 2012). The potential of DNA metabarcoding applications is vast, with the ability to quickly determine the species composition of practically any sample (Dormontt et al. 2018). Thus, DNA metabarcoding of the primates’ diet in this study is the most suitable technique to provide powerful insights into the diet variation between *S.syndactylus* and *M.nemestrina* by generating a list of plants eaten by both species in the highland area. Data from the diet composition will contribute to our knowledge of feeding patterns and foraging techniques (Zhang et al. 2024). Therefore, the objectives of this study were to assess and compare the dietary preferences and to determine the presence of interspecific competition between *S.syndactylus* and *M.nemestrina*, based on their diet intake.

## Material and methods

### Faecal sample collection

**Figure 2.**
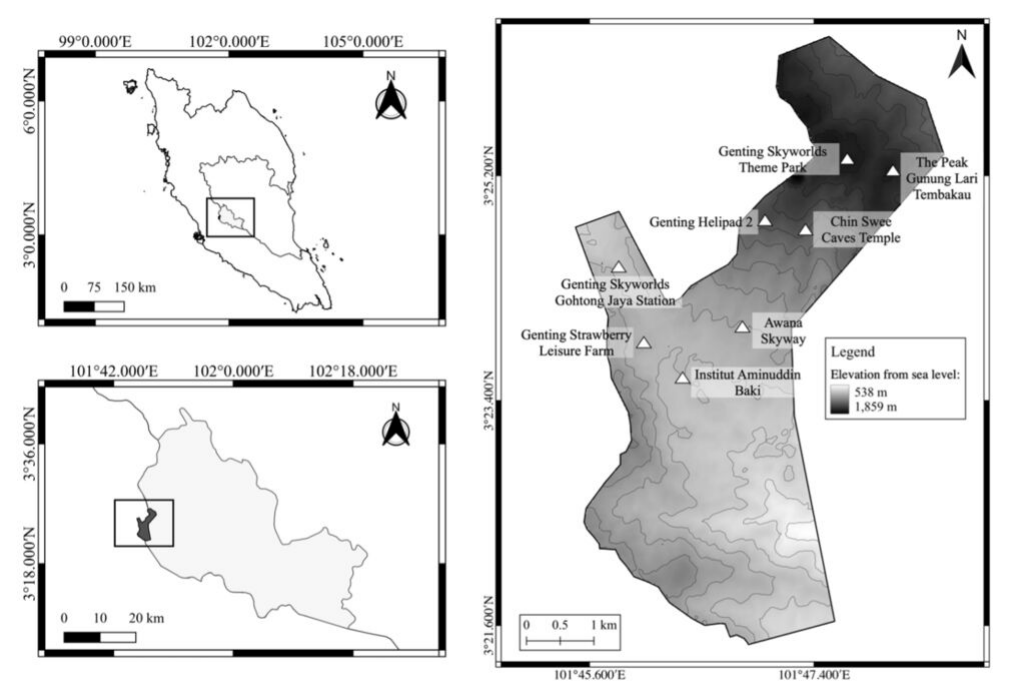
The map of Genting Highlands.

All analysed samples (n = 8) in the present study were collected from non-invasive faecal material sampled at the Genting Highlands, Pahang (3°25′25″N, 101°47′36″E) (Table 1, Fig. 2). Sample collection for *S.syndactylus* was conducted from March 2021 until June 2022, while M.nemestrina was collected in October 2020. Only one individual of *M.nemestrina* was used in this study because of the limited sample availability during faecal collection at the Genting Highlands. Thus, the findings from this research are still at a preliminary stage. The samples were collected from the core of the faecal mass to avoid including other contaminants (Gani et al. 2024). The collected faecal samples were placed into sterile 45-ml tubes and fixed with 95% ethanol for long-term storage (Khairulmunir et al. 2023). All samples were labelled and stored at -20°C. The faecal samples were genetically identified using mitochondrial DNA D-loop region sequences and confirmed using NCBI GenBank BLASTn.

### Library preparation and sequencing of trnL amplicons

DNA was extracted from approximately 400 mg of each faecal sample using the innuPREP Stool DNA Kit (Analytik Jena, Jena, Germany) according to the manufacturer’s instructions. The quality of the purified DNA was assessed on a 1% TAE agarose gel and the DNA concentration was measured using a spectrophotometer (Implen NanoPhotometer® N60/N50) and via fluorometric quantification using an iQuant™ Broad Range dsDNA Quantification Kit. Eight purified genomic DNA (gDNA) samples with good DNA quality were subjected to library preparation for trnL gene amplicon sequencing. The purified gDNA was amplified using locus-specific sequence primers of the trnL gene with overhang adapters (trnL-forward: 5′ TCGTCGGCAGCGTCAGATGTGTATAAGAGACAG‐[GGGCAATCCTGAGCCAA] 3′ and trnL-reverse: 5′ GTCTCGTGGGCTCGGAGATGTGTATAAGAGACAG-[CCATTGAGTCTCTGCACCTATC] 3′) (Taberlet et al. 2007). Library amplification was performed using KOD-Multi & Epi-® (Toyobo). Dual indices were attached to the amplicon PCR using the Illumina Nextera XT Index Kit version 2, according to the manufacturer’s protocols. The quality of the libraries was measured using an Agilent Bioanalyzer 2100 System with an Agilent DNA 1000 Kit and via fluorometric quantification using Helixyte Greenä Quantifying Reagent. The library was normalised and pooled according to the protocol recommended by Illumina, and 150-paired-end sequencing was performed using the MiSeq platform.

### Data processing and statistical analysis

Raw FASTQ data from the eight samples were filtered and assessed using fastqc (https://www.bioinformatics.babraham.ac.uk/projects/fastqc/). Subsequently, amplicon sequence variant (ASV) data were produced through a process that included filtering, denoising, merging and removing chimeras using DADA2 (Callahan et al. 2016). Taxonomic assignment was performed against the NCBI GenBank database. The resulting ASV data were then imported and subjected to dietary characterisation analysis using R Studio (version 2023.09.1).

## Data resources

All next-generation sequencing data have been uploaded to the National Center of Biotechnology Information (NCBI) under the Sequence Read Archive Bioproject accession number PRJNA1073585. The biosample accession numbers corresponding to each of the samples are listed in Table 1.

## Results

### Sequencing bioinformation analysis and dietary characterization

The Illumina MiSeq sequencing run successfully generated 756,663 raw reads for the trnL marker from the eight gDNA samples. The average number of reads per sample ranged from 65,723–105,212. Table 1 lists the total number of raw reads, followed by the filtered, denoised and merged data, with chimera sequences removed. The final non-chimeric sequences consisted of 640,991 reads that were used for diet profiling analyses, which resulted in 140 ASVs (Table 1). From the total non-chimeric reads, the taxonomic classification indicated that 32.5% of the trnL sequences could not be assigned to any taxonomic group (unknown/unidentified sequences).

**Figure 3.**
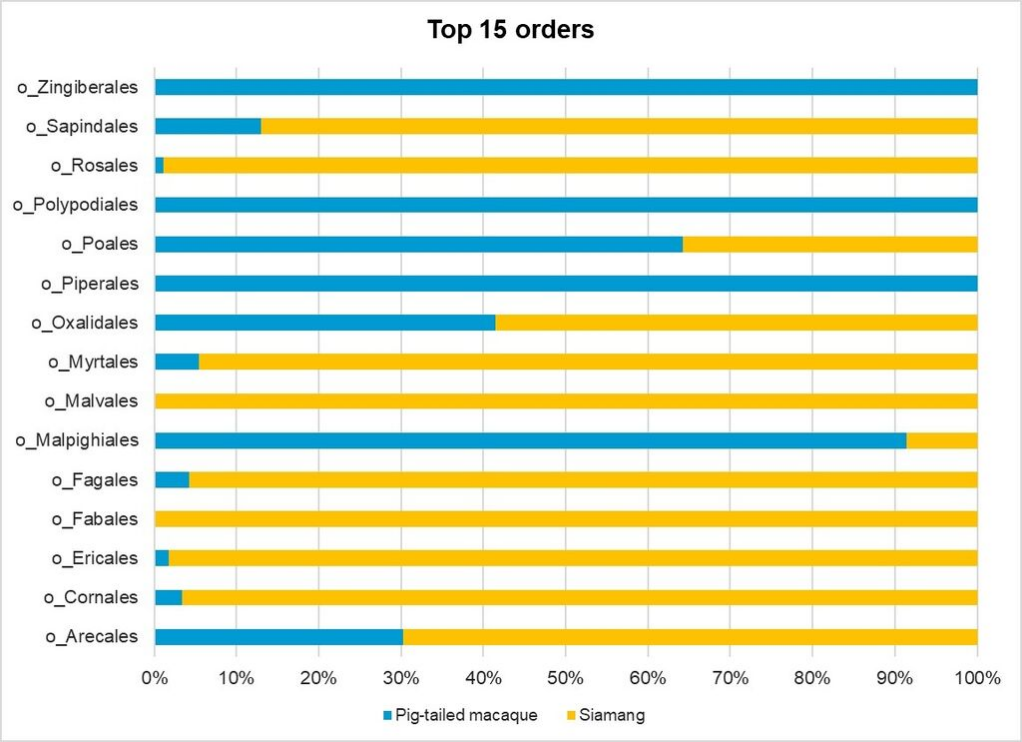
Abundance of the top 15 plant taxa at the order level that were identified in the faecal samples of *Symphalangussyndactylus* (siamang) and *Macacanemestrina* (pig-tailed macaque).

The 140 ASVs obtained featured 26 orders, 46 families, 60 genera and 49 species that were identified from 23 classes. Both *S.syndactylus* and *M.nemestrina* exhibited different plant dietary intakes that were identified in 23 and 15 orders, respectively. The most prevalent plant orders identified as being consumed by *S.syndactylus* were Fabales (33.37%), Rosales (17.58%), Asterales (4.74%), Myrtales (3.69%) and Ericales (2.13%). Meanwhile, the most abundant order identified as being consumed by *M.nemestrina* was Piperales (51.25%), followed by Arecales (23.51%), Oxalidales (2.6%), Polypodiales (2.51%) and Myrtales (2.44%). Fig. 3 shows the top 15 identified plants at the order level.

**Figure 4.**
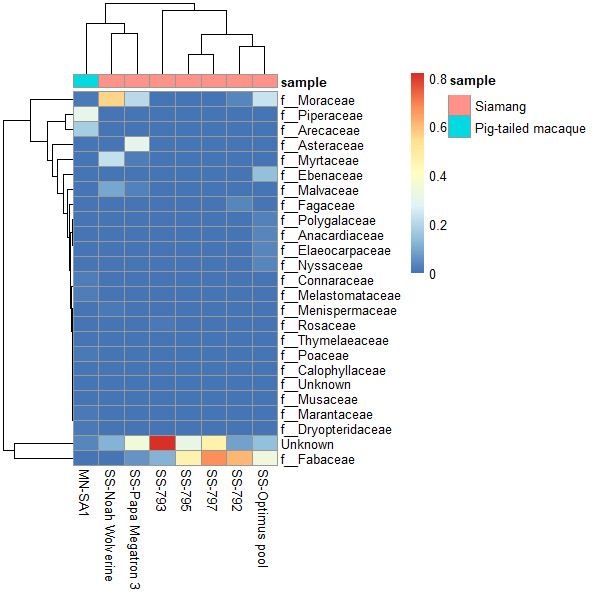
Heatmap of the top 25 plant taxa at the family level in *Symphalangussyndactylus* (siamang) and *Macacanemestrina* (pig-tailed macaque).

**Figure 5.**
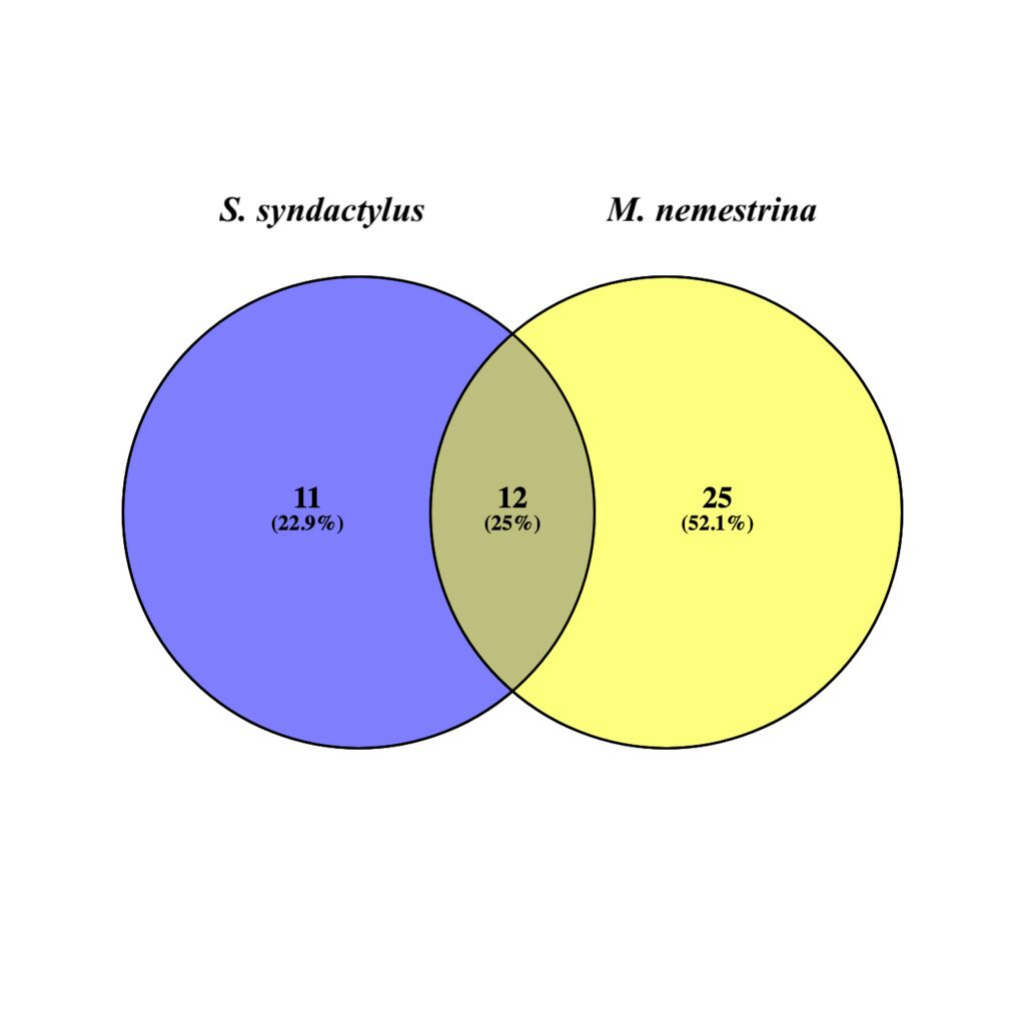
The number of unique and shared ASVs between *Symphalangussyndactylus* (siamang) and *Macacanemestrina* (pig-tailed macaque).

Taxonomy classification results showed differences in the prevalent plant taxa of the diets between *S.syndactylus* and *M.nemestrina*. *Symphalangussyndactylus* displays a similar pattern of plant preferences (Fig. 4). In *S.syndactylus* samples, the Fabaceae family exhibited the highest abundance at 32.9%, followed by Moraceae (17.43%), Asteraceae (4.74%), Myrtaceae (3.67%) and Ebenaceae (2.13%). Meanwhile, the most prevalent family species in the diet intake of *M.nemestrina* were identified as Piperaceae (51.25%), Arecaceae (23.51%), Connaraceae (2.6%), Melastomataceae (2.44%) and Moraceae (2.31%). The Venn diagram below illustrates both the number and percentage of the shared core ASVs between the *S.syndactylus* and *M.nemestrina* samples. Of the total ASVs detected, 48 exhibited an exclusive composition in all samples at the family level, whereas 11 ASVs were unique to *S.syndactylus* and 25 ASVs belonged to *M.nemestrina*. Interestingly, 12 ASVs were common to both *S.syndactylus* and *M.nemestrina* (Fig. 5).

**Figure 6.**
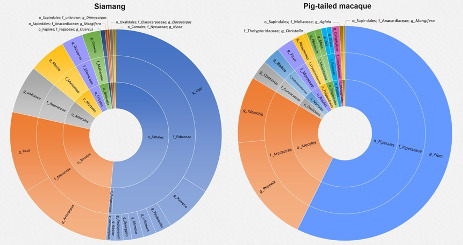
Taxonomic composition of the most abundant taxa at the order, family and genus levels in *Symphalangussyndactylus* (siamang) and *Macacanemestrina* (pig-tailed macaque).

The sunburst chart displays the taxonomic composition and relative abundance of the most prevalent taxa at the order, family and genus levels from the faecal samples of both *S.syndactylus* (siamang) and *M.nemestrina* (pig-tailed macaque) (Fig. 6). Each ring is segmented proportionally to convey its relative taxonomic abundance. The order Fabales represents the most abundant plant's genus in the S.syndactylus faecal sample. The primary genera found in the Fabales order were *Inga*, followed by *Pueraria*, *Paubrasilia*, *Mimosa*, *Senegalia*, *Pediomelum*, *Glycine* (soybean) [Family: Fabaceae] and *Xanthophyllum* [Family: Polygalaceae]. Other prominent genera identified in the *S.syndactylus* sample include *Artocarpus* (terap), *Ficus* (common fig), *Myrcia*, *Diospyros* (kayu arang), *Grewia* (chenderai), *Quercus* (oak) and *Mangifera* (mango). The dominant genus identified in the faecal samples of *M.nemestrina* included *Piper* (pepper), *Phoenix* and *Mauritia* (palm), *Connarus* (woody climber), *Pleocnemia* and *Christella* (fern), *Blakea* (senduduk), *Phrynium* (arrowroot), *Musa* (banana), *Ficus* (common fig), *Oryza* (rice), *Zea* (corn), *Calophyllum* (bintangor), *Mangifera* (mango) and *Aglaia* (bekak). From the listed plant genera, both *Paurasilia* and *Pediomelum* were found to be native from other continental. The identification of these plant genera were based on similarities in DNA sequences with actual plants that are not included in the database. Plants are assigned to the nearest genus when there is low or virtually identical sequence variation, which also contributes to the conflict in diet identification using the trnL gene region (Valentini et al. 2009). Furthermore, only six species of plants from the 60 identified genera were consumed by both *S.syndactylus* and *M.nemestrina* in the Genting Highlands, which included *Blakea* sp., *Diospyros* sp., *Ficus* sp., *Glycine* sp., *Mangifera* sp. and *Xanthophyllum* sp.

## Discussion

Overall, this study revealed that Fabaceae and Moraceae (*Artocarpus* sp. and *Ficus* sp.) were the preferred diets consumed by *S.syndactylus* in the Genting Highlands, which accounted for 50.33% of the total diet, with Fabaceae as the most diverse diet. Based on a previous study on *S.syndactylus* in the Genting Highlands, nine species of plants were identified in their diet, including *Ardisiacrispa*, *Caryotamitis*, *Duabangagrandiflora*, *Ficusbenjamina*, *Ficusracemosa*, *Ficusseptica*, *Heritierasumatrana*, *Piperaduncum* and *Syzygiumcampanulatum* (‌Muhd-Sahimi et al. 2020), with the fruits from *Ficus* sp. being the most consumed plant. However, no traces of these plant species were detected in this study, except for *Ficus* spp. Generally, *S.syndactylus* in the wild primarily feeds on fruits (49%) and leaves (38%). In addition, they include flowers and insects in their diet in smaller percentages (Palombit 1992, Palombit 1997, Bartlett 2007). Compared to its sister genus, *Hylobateslar*, which prefers pulpy fruit trees and lianas, *S.syndactylus* prefers immature foliage, mainly from lianas, because it provides more young leaves compared to other trees (Palombit 1997). Meanwhile, for *M.nemestrina* in the Genting Highlands, Piperaceae (*Piper* sp.) was the most abundant plant consumed, followed by Arecaceae (*Phoenix* sp. and *Mauritia* sp.), which accounted for 74.76% of its total diet. Unlike its sister species, the family Fabaceae and Moraceae is the preferred diet for both *Macacafascicularis* and *Macacaartoides* (Osman et al. 2020, Osman et al. 2022). However, notably, *M.nemestrina* in the Genting Highlands also preferred *Ficus* sp., which is a similar preferred diet for both *M.fascicularis* and *M.arctoides*. According to Phillipps and Phillipps (2018), 90% of the food intake of *M.nemestrina* is fruit. In addition, because of the many oil palm plantations along the edge of forests in Borneo, oil palm fruits appear to replace figs as the fallback diet (Phillipps and Phillipps 2018).

Genting Highlands is a place that embodies nature, entertainment, hospitality and tourism. It harbours primate communities from the families Hylobatidae and Cercopithecidae (Md-Zain et al. 2021), such as *S.syndactylus* (siamang), *T.obscurus* (dusky langur), *P.siamensis* (pale-thighed langur), *M.nemestrina* (pig-tailed macaque) and *M.fascicularis* (long-tailed macaque), which have been reported to co-exist with one another within the same area (Palombit 1992, Gron 2008, Md-Zain et al. 2021). Overlapping of niche resources may occur if the primate density in the area increases (Md-Zain et al. 2021). Based on the findings of this study, it is suggested that little interspecific competition in diet was detected between Hylobatidae (*S.syndactylus*) and Cercopithecidae (*M.nemestrina*), with only 10% shared plant genera in their diet. In terms of plant similarities, only 10 plants from 46 families were consumed by both species, including Anacardiaceae, Arecaceae, Euphorbiaceae, Fabaceae, Fagaceae, Melastomataceae, Moraceae, Poaceae and Polygalaceae. Congruent with the previously reported diet of Hylobatidae and Cercopithecidae by Barus et al. (2018), which was conducted at the Ape Park Tourist Area Forest of North Sumatera, Arecaceae, Euphorbiaceae, Fagaceae and Moraceae were amongst the common shared plants between *S.syndactylus* and *M.nemestrina*. Apart from their diet similarities, the findings of this study further revealed that, out of the 46 families identified using the trnL marker barcoding, 25 families were not present in *S.syndactylus*. The families Piperaceae, Connaraceae and Dryopteridaceae were absent in *S.syndactylus*, which suggests that they did not consume species from these families. However, Asteraceae and Myrtaceae were not detected in *M.nemestrina*. Therefore, it can also be concluded that the mixed association of these species in the Genting Highlands does not significantly affect their feeding activities.

*Ficus* sp. (Moraceae) was the only plant amongst the top five preferred diets consumed by both *S.syndactylus* and *M.nemestrina* in this study. In particular, *S.syndactylus* spends twice as much of their feeding activity consuming fig fruits (*Ficus* spp.; Moraceae) (Palombit 1997). The majority of primates in the Genting Highlands commonly ate fig trees (*Ficusbenjamina*) and nearly 80% of the Genting Highland primates rely on these trees for sustenance (Md-Zain et al. 2021). As both *S.syndactylus* and *M.nemestrina* have a similarly high preference for *Ficus* sp., it is predicted that these primates may exhibit niche differentiation behaviour, either spatial-, trophic- or time-dependent, to decrease any form of interspecific competition between them. Based on the niche partitioning hypothesis, heterogeneity in food and habitat utilisation amongst group members reduces competition between conspecific, cohesively grouped species (Sheppard et al. 2018). This was observed by Md-Zain et al. (2021), where members of Cercopithecidae in the Genting Highlands were simultaneously observed foraging for fruits from a fig tree. However, these Cercopithecidae members dispersed when *S.syndactylus* started to roam the area to forage. Furthermore, *S.syndactylus* is a strictly arboreal species, using every canopy level that is commonly found in highland areas. Unlike *S.syndactylus*, *M.nemestrina* is a terrestrial primate that feeds on scarce fruiting trees (Phillipps and Phillipps 2018) and spends most of its time in the lower canopy (Bowles 1989). *Macacanemestrina* only forages in the upper canopy to consume large fruiting trees (Bowles 1989). The behavioural differences in their niche elevation may have allowed them to share similar fig trees in the Genting Highlands.

Overlapping of niches was also observed in other species of Hylobatidae (*Hoolockhoolock*) and Cercopithecidae (*Macacaleonine*) at Satchari National Park, situated within the Raghunandan Hill Reserve Forest, Bangladesh. A study by Neha et al. (2021) presented similar findings amongst the shared plants in common between both families. The diet competition between *H.hoolock* and *M.leonina* was intense because more than 50% of their total diet consisted of shared fruit plants (25 species). In addition, it has been noted that, by specialising in various food sources and forest patches, *H.hoolock* generally reduces interspecific competition with other species (Neha et al. 2021). These interactions would putatively explain how members of Hylobatidae can co-exist with members of Cercopithecidae in the same area.

The elucidation of the diet of wild primates has allowed researchers to compare the dietary niches of various species that co-exist within a shared habitat (Tutin and Fernandez 1993). The distribution and accessibility of food resources play a significant role in determining ecological variation between different primate species. The Genting Highland flora represents a rarely dispersed higher montane cloud forest and elfin woodland in Peninsular Malaysia (Stone 1981). Although the surroundings ecosystems may have been affected by current development, the habitat areas are considered to be preserved as virgin forest (Azmi 2021). Notably, this study found that both *S.syndactylus* and *M.nemestrina* are generalist frugivores because their main intake in the Genting Highlands is a varied, fruit-dominant diet. In fact, *M.nemestrina* is known to be the most frugivorous species compared to other *Macaca* spp. (Caldecott 1986). Nevertheless, based on this study, the dietary preferences of *S.syndactylus* and *M.nemestrina* differed significantly in the Genting Highlands. Both species displayed a high preference for various fruiting trees, thus indicating that the flora of this highland area is still well preserved. The diversity of the diet between these two families suggests that primates in the Genting Highlands are not affected by changes in their surroundings.

## Conclusions

The application of DNA metabarcoding in faecal samples to determine the diet of *S.syndactylus* and *M.nemestrina* identified 60 genera and 49 species of plants. A comparison of the diets between Cercopithecidae and Hylobatidae revealed only minor similarities in plant consumption, which suggests that both groups have different diet preferences provided that food sources are not scarce. The diversity of diet preferences for fruiting trees also suggests that these primates are not significantly affected by the current development in the Genting Highlands. Although the data for *M.nemestrina* are derived from a single individual, current findings on their diet can be used as fundamental data and estimations for future study. Amongst the six genera consumed by both species, *Ficus* sp. had the highest prevalence. Niche differentiation behaviour may have been performed by the primates to prevent interspecific competition for *Ficus* sp. Due to the lack of published studies, especially regarding primates in highland areas such as the Genting Highlands, preliminary diet data from this study are necessary as a basic guideline and for future conservation management purposes by the responsible authorities. Therefore, due to the limitation of the small sample size in this study, a more comprehensive study is highly recommended in the future to further elucidate the diet amongst the primate communities, Hylobatidae and Cercopithecidae in the Genting Highlands. This include the feeding heights and substrate analysis; collection and analysis of more faecal samples of different season and factors that affect its variability diet; and development of a local plant database in Malaysia to support diet identification in primates.

## Figures and Tables

**Table 1.** High-throughput sequence statistics were generated using DADA2 V1.18 tools.

**Species**	**Sample**	**Siamang Group**	**Accession No.**	**Raw reads**	**Filtered**	**Non-chimeric**
*S.syndactylus*	SS-792	Socrates	SRR27881821	88,948	67,209	66,298
	SS-793	Socrates	SRR27881884	99,630	86,235	85,482
	SS-795	Socrates	SRR27881963	105,212	69,579	68,052
	SS-797	Socrates	SRR27881964	103,832	101,585	98,986
	SS-Noah Wolverine	Wolverine	SRR27882037	99,578	97,473	94,750
	SS-Optimus pool	Optimus	SRR27882052	94,819	92,667	86,512
	SS-Papa Megatron 3	Megatron	SRR27882068	98,921	96,947	89,466
*M.nemestrina*	NC13		SRR27883313	65,723	56,679	51,445
	**TOTAL**			**756,663**	**668,374**	**640,991**
